# Photon-Counting CT Scan Phantom Study: Stability of Radiomics Features

**DOI:** 10.3390/diagnostics15060649

**Published:** 2025-03-07

**Authors:** Lama Dawi, Kodjodenis Amouzouga, Serge Muller, Cyril Nallet, Arnaud Dupont, Benoit Vielliard, Cedric Croisille, Aurelie Moussier, Gabriel Garcia, François Bidault, Remy Barbe, Salma Moalla, Thibaut Pierre, Corinne Balleyguier, Jules Dupont, Nathalie Lassau

**Affiliations:** 1 Radiology Department, Gustave Roussy, 94805 Villejuif, France; 2 Laboratoire d’Imagerie Biomedicale Multimodale Paris-Saclay (BioMAPS), Université Paris-Saclay, Inserm, Centre National de la Recherche Scientifique, Commissariat à l’Energie Atomique, 94800 Villejuif, France; 3 Department of Computed Tomography, Siemens Healthineers AG, 91301 Forchheim, Germany; 4 Faculty of Medicine, Université Paris-Saclay, Le Kremlin Bicètre, 94270 Paris, France

**Keywords:** photon-counting CT scan, radiomics, phantom study

## Abstract

**Background/Objectives**: To evaluate and optimize the reconstruction parameters of images acquired with a photon-counting CT scanner to achieve a stable radiomics signal. **Methods**: Radiomics is a quantitative imaging biomarker correlated to survival in oncology patients. Implementing radiomics in clinical routine remains challenging due to the feature’s instability. Photon-counting CT scans use innovative technology directly converting photons into electrical signals resulting in higher-resolution images with reduced artifacts. This study used two organic phantoms: a natural wet sponge and a dry sausage. UHR images were acquired using a NAEOTOM Alpha photon-counting CT scan (Siemens) with a 0.4 mm slice thickness and 0.3 × 0.3 mm pixel size. Tube current and voltage were fixed at 112 mA and 120 KvP. A total of 24 reconstruction parameter sets were obtained by combining different values of kernel (Br), quantitative iterative reconstruction (QIR), spectral reconstruction (keV), and matrix size. Ten successive acquisitions were obtained on both phantoms. In total, 93 radiomic features were extracted on an ROI using the default parameters of Pyradiomic 3.0.1. Each feature’s stability was evaluated using the coefficient of variation (CV) within each parameter set. **Results**: Of the 24 reconstruction parameter sets, 5 were selected based on best image quality by seven radiologists and three radiology technologists. Radiomics features were considered stable on a set when CV was less than 15%. Feature stability was impacted by reconstruction parameters and the phantom used. The most stable combination included 90 and 65 stable features of the 93 tested on the sausage and sponge respectively. It was configured with Br36, QIR 4, 60 keV, and a 1024 × 1024 matrix size. **Conclusions**: Images obtained on photon-counting CT scans offer promising radiomic feature stability with optimal parameter configurations that could be applied in a clinical setting.

## 1. Introduction

Photon-counting detector CT scans (PCD-CT) represent a significant advancement in medical imaging, utilizing a novel detector technology that directly measures individual X-ray photons and converts them to an electrical signal [1]. Among other advantages are improved spatial resolution with no dose increase, availability of multi-energy imaging data, and artifact and noise reduction [2].

Clinical research on PCD-CT has proved their superiority over standard and dual-energy CT scans in phantom studies [3] and clinical applications such as cardiac imaging [4], where sensitivity in detecting coronary artery disease exceeded 90% [5]. Applications in oncology are still limited but ongoing research in radiomics is promising [6,7].

Radiomics is the translation of images into quantitative minable data allowing the interpretation of textures beyond the capacity of the human eye [8]. This data provides valuable prognostic and predictive information, which could lead to identifying noninvasive biomarkers [6,7,8,9,10,11]. This is especially true with the emerging use of immunotherapy in oncology where traditional diagnostic and treatment evaluation methods are insufficient [12]. In a prospective study done on 575 patients, Dercle et al. found that radiomics and machine learning can analyze data from routine computed tomography scans to enhance clinical decision-making for melanoma patients undergoing immunotherapy [13]. Despite previous and ongoing studies, the implementation of radiomics in clinical routine is problematic due to feature reproducibility, stability, and standardization [14,15,16]. Many studies demonstrated how radiomics feature reproducibility is influenced by acquisition parameters [17], reconstruction parameters [18], Ct techniques [19], region of interest position [20,21,22], and tumor segmentation [23], among other imaging factors.

We conducted a phantom-based study to optimize the reconstruction parameters of a PCD-CT scanner operating in UHR mode. This optimization aimed to provide high-resolution CT scan images suitable for routine clinical evaluation while ensuring a robust and repeatable radiomics signal to extract future imaging biomarkers.

## 2. Materials and Methods

### 2.1. Phantom Characteristics and Imaging Parameters

To assess the heterogeneity of tissues on phantoms, we selected two different homemade objects for our experiments. The first object was a natural sea sponge inside a plastic bag filled with water (phantom 1). The second object was a dry sausage (phantom 2). Both phantoms provide images with a significant spatial heterogeneity level suitable for radiomics analysis.

Images used in this study were acquired on a photon-counting CT scanner (NAEOTOM Alpha^®^, Siemens Healthineers, Erlangen, Germany). The detector of this CT scanner directly converts X-rays into an electrical signal, which is then used to create an image with an in-plane spatial resolution of up to 0.11 mm. Image acquisitions were conducted in UHR mode with a tube voltage of 120 kV, a collimation of 120 × 0.2 mm, and a spatial resolution of 0.16 × 0.11 × 0.11 mm. We operated at a CDTIvol of 9 mGy to comply with the latest diagnostic reference levels in medical imaging established by the International Commission on Radiological Protection [24].

In the first step, we explored the parameter space of reconstructed image rendering by varying the following parameters:

- Spectral reconstruction mono-energy: {40, 60} keV
- Reconstruction kernel: {Br36, Br40}
- Quantum Iterative Reconstruction (QIR) strength: {2, 3, 4}
- Matrix size: {512 × 512, 1024 × 1024}

### 2.2. Parameters Combination and Set Selection

The twenty-four possible combinations of the four parameters mentioned above were considered (Table 1). The 24 images of phantom 2 (dry sausage) were presented to a group of ten medical imaging professionals (seven radiologists and three technicians). They reviewed the images blindly and applied a forced-choice ranking to select the 5 best-reconstructed images based on image quality, defined as a mix of perceived spatial resolution, contrast resolution, and signal-to-noise ratio. The mean values of the forced-choice ranks over the 10 readers were used to identify the five sets of reconstruction parameters leading to the best-perceived image quality (the higher the rank value, the better the perceived image quality) (Table 2). Finally, the five sets of reconstruction parameters selected to evaluate the robustness of the radiomics texture characteristics were as follows:

- Set 1: Br36, QIR 4, 1024 × 1024, 60 keV
- Set 2: Br36, QIR 4, 1024 × 1024, 40 keV
- Set 3: Br36, QIR 4, 512 × 512, 40 keV
- Set 4: Br40, QIR 4, 1024 × 1024, 40 keV
- Set 5: Br40, QIR 4, 512 × 512, 40 keV

**Table 1:** Sets of reconstruction parameters considered in this study.

40 keV	60 keV
Br36, QIR 2, 512 × 512	Br36, QIR 2, 512 × 512
Br36, QIR 3, 512 × 512	Br36, QIR 3, 512 × 512
Br36, QIR 4, 512 × 512	Br36, QIR 4, 512 × 512
Br36, QIR 2, 1024 × 1024	Br36, QIR 2, 1024 × 1024
Br36, QIR 3, 1024 × 1024	Br36, QIR 3, 1024 × 1024
Br36, QIR 4, 1024 × 1024	Br36, QIR 4, 1024 × 1024
Br40, QIR 2, 512 × 512	Br40, QIR 2, 512 × 512
Br40, QIR 3, 512 × 512	Br40, QIR 3, 512 × 512
Br40, QIR 4, 512 × 512	Br40, QIR 4, 512 × 512
Br40, QIR 2, 1024 × 1024	Br40, QIR 2, 1024 × 1024
Br40, QIR 3, 1024 × 1024	Br40, QIR 3, 1024 × 1024
Br40, QIR 4, 1024 × 1024	Br40, QIR 4, 1024 × 1024

**Table 2:** Evaluation of reconstructed images (phantom 2) by 10 readers using a forced-choice ranking of the top 5 images (the higher the rank value, the better the perceived image quality). Highlighted lines correspond to the 5 highest scores.

Reconstruction Sets	Reader A	Reader B	Reader C	Reader D	Reader E	Reader F	Reader G	Reader H	Reader I	Reader J	Score (Mean)	Preferred Sets
60 keV, Br36, QIR2, 1024 × 1024	0	0	0	0	0	0	0	0	0	0	0.00	
60 keV, Br36, QIR3, 1024 × 1024	1	0	1	0	0	0	0	0	0	2	0.4 0	
60 keV, Br36, QIR4, 1024 × 1024	3	0	4	5	0	0	0	0	0	1	1.3 0	Set 1
60 keV, Br36, QIR2, 512 × 512	0	0	0	0	0	0	0	0	0	0	0.00	
60 keV, Br36, QIR3, 512 × 512	4	0	0	2	0	0	0	0	0	0	0.6 0	
60 keV, Br36, QIR4, 512 × 512	0	0	3	0	0	0	0	0	1	0	0.4 0	
60 keV, Br40, QIR2, 1024 × 1024	0	0	0	0	0	0	0	0	0	5	0.5 0	
60 keV, Br40, QIR3, 1024 × 1024	0	0	0	0	0	0	0	0	0	4	0.4 0	
60 keV, Br40, QIR4, 1024 × 1024	2	0	0	3	0	0	0	0	0	3	0.8 0	
60 keV, Br40, QIR2, 512 × 512	0	0	0	0	0	0	0	0	0	0	0.00	
60 keV, Br40, QIR3, 512 × 512	0	0	0	0	0	0	0	0	0	0	0.00	
60 keV, Br40, QIR4, 512 × 512	5	0	0	4	0	0	0	0	0	0	0.9 0	
40 keV, Br36, QIR2, 1024 × 1024	0	0	0	0	2	1	0	0	0	0	0.3 0	
40 keV, Br36, QIR3, 1024 × 1024	0	0	0	0	3	0	0	0	0	0	0.3 0	
40 keV, Br36, QIR4, 1024 × 1024	0	5	2	0	5	0	0	4	4	0	2.00	Set 2
40 keV, Br36, QIR2, 512 × 512	0	0	0	0	4	0	2	0	0	0	0.6 0	
40 keV, Br36, QIR3, 512 × 512	0	1	0	1	0	0	0	0	0	0	0.2 0	
40 keV, Br36, QIR4, 512 × 512	0	4	0	0	0	0	4	0	5	0	1.3 0	Set 3
40 keV, Br40, QIR2, 1024 × 1024	0	0	0	0	0	5	0	0	0	0	0.5 0	
40 keV, Br40, QIR3, 1024 × 1024	0	0	0	0	1	4	0	1	0	0	0.6 0	
40 keV, Br40, QIR4, 1024 × 1024	0	3	5	0	0	0	3	5	2	0	1.8 0	Set 4
40 keV, Br40, QIR2, 512 × 512	0	0	0	0	0	3	0	0	0	0	0.3 0	
40 keV, Br40, QIR3, 512 × 512	0	0	0	0	0	2	1	2	0	0	0.5 0	
40 keV, Br40, QIR4, 512 × 512	0	2	0	0	0	0	5	3	3	0	1.3 0	Set 5

### 2.3. Radiomics Analysis

**Figure 1:**
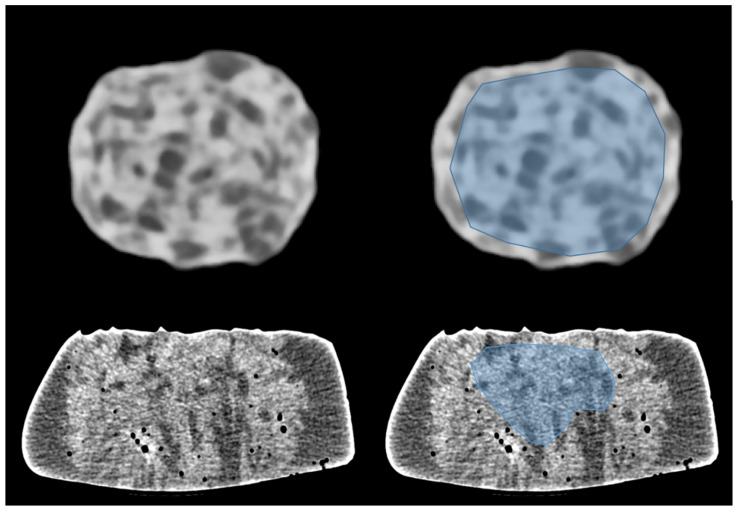
Images of phantom 1 (**top**) and phantom 2 (**bottom**) acquired in UHR mode. Regions of interest (ROI) were drawn to exhibit heterogeneity in density (blue area on right images). ROI include the inner part of the dry sausage (**top-right**). ROI exclude air bubbles in the wet sponge (**bottom-right**).

In a second step, we performed a radiomics analysis on 10 different acquisitions of both phantom 1 and phantom 2 with the 5 previously selected sets of reconstruction parameters. Our intent was to compare the repeatability of radiomics features between the two phantoms, and to determine if a specific set of reconstruction parameters was leading to a better repeatability of radiomics features. All images were acquired in UHR mode and reconstructed with the five sets of reconstruction parameters. Then, regions of interest (ROI) exhibiting heterogeneity in density were segmented (Figure 1) using 3D Slicer software (version 5.6.2). ROI masks and images were then processed with Pyradiomics (version 3.0.1) to extract their 93 textural features (18 first-order features and 75 second-order features). Second-order features included measures on the gray level co-occurrence matrix (glcm), the gray level dependence matrix (gldm), the gray level size zone matrix (glszm), the gray level run length matrix (glrlm), and the neighboring gray tone difference matrix (ngtdm). The repeatability of the features was assessed using the coefficient of variation (CV), defined as follows:

(1) CV=σμ

where *σ* corresponds to the standard deviation of the measurements over the same reconstructed slice from the ten acquisitions and *μ* to the average value of these measurements. In this study, we set a threshold on CV equal to 15%, under which we considered the repeatability of the radiomics features acceptable in clinical routine.

For each set of reconstruction parameters, a comparison of proportions was performed to look for differences in the percentage of stable radiomics features between phantom 1 and phantom 2. A total of *N*-1 Chi-square tests were used, and confidence intervals were calculated and *p*-values were derived to assess the level of statistical significance of proportion differences.

For each phantom, a multiple regression analysis was performed using the proportion of stable radiomics features over the five sets of reconstruction parameters. The response was the fraction of stable radiomics features (*Y*) observed in images acquired with the 5 sets of reconstruction parameters, while the predictors were the convolution kernel (*X*_1_), the reconstruction matrix size (*X*_2_), and the mono-energy value (*X*_3_). To perform this multiple regression, we coded the two possible choices of each of these predictors with numerical values (Table 3 and Table 4 for phantom 1 and phantom 2, respectively). This analysis allowed us to identify which reconstruction parameters had the highest impact on the percentage of stable radiomics features for both phantoms. We computed the *p*-values to assess the statistical significance of each coefficient of the regression. The *p*-values were calculated for each coefficient of regression with the null hypothesis being that the coefficient is equal to zero. A low *p*-value (<0.05) means that the coefficient is likely not to equal zero, and therefore suggests that the feature has a significant impact on the percentage of stable radiomics features. A high *p*-value (>0.05) means that we cannot conclude that the considered reconstruction parameter affects the percentage of stable radiomics features. Finally, we used the R-squared value as a measure of how well the regression model explained the percentage of stable radiomics features.

## 3. Results

### 3.1. Stability of Radiomics Features

**Figure 2:**
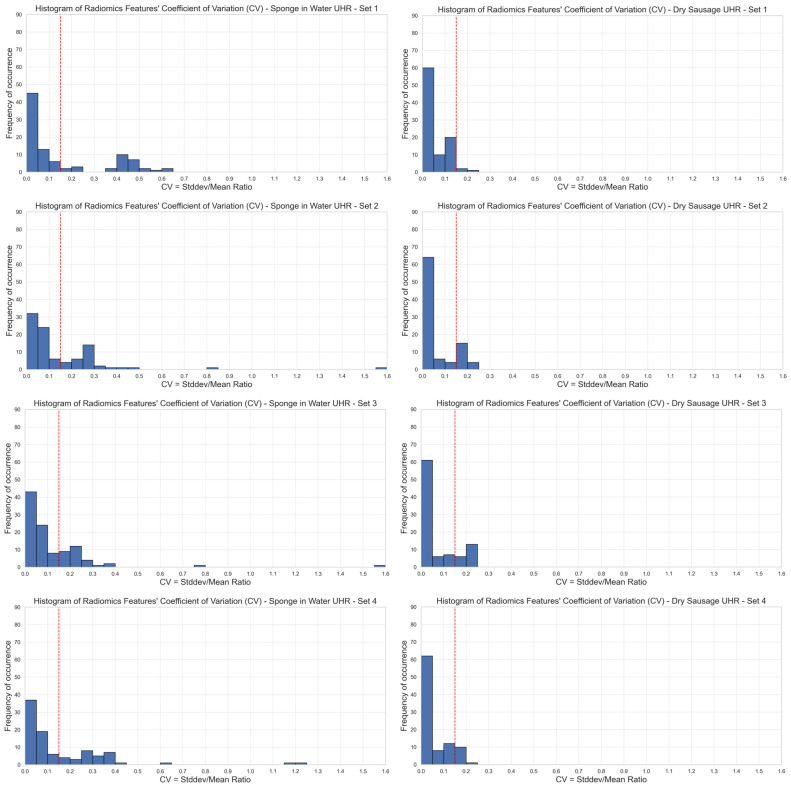

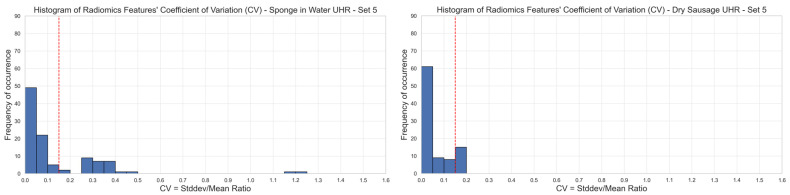
Stability of radiomics features illustrated through the distribution of coefficients of variation (CV). Each blue bar corresponds to the occurrence of CV values within a CV interval of 0.05. The red line corresponds to a 0.15 CV threshold. Stable features have a CV lower than 0.15 (**left**: phantom 1, **right**: phantom 2, **top-to-bottom**: sets {1,2,3,4,5} of reconstruction parameters). UHR mode.

Based on our 15% CV threshold criterion, we observed that only 67–70% of the radiomics features were stable over different image acquisitions of phantom 1 (sponge in water), depending on the set of reconstruction parameters that was selected. We observed 65/93 stable features with set 1 (62/93, 63/93, 62/93, and 64/93 for set 2, set 3, set 4, and set 5, respectively). Among the five sets of reconstruction parameters, we had a group of 60 stable features in common (Figure 2, left). The first-order radiomics features were the most stable with 94% (17/18) stable features among the five sets of reconstruction parameters.

Regarding phantom 2 (dry sausage), we observed that 80–98% of the radiomics features were stable over the ten image acquisitions, depending on the set of reconstruction parameters that was selected. We observed 90/93 stable features with set 1 (74/93, 74/93, 82/93, and 82/93 for set 2, set 3, set 4, and set 5, respectively). Among the five sets of reconstruction parameters, we had a group of 71 stable features in common (Figure 2, right). All first-order radiomics features (18/18) were stable among the five sets of reconstruction parameters.

**Figure 3:**
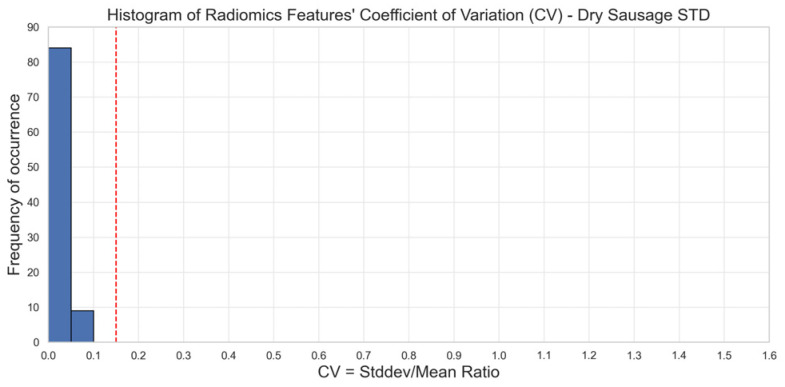
Stability of radiomics features illustrated through the distribution of coefficients of variation (CV). Each blue bar corresponds to the occurrence of CV values within a CV interval of 0.05. The red line corresponds to a 0.15 CV threshold. Stable features have a CV lower than 0.15 (phantom 2). STD mode. We observe that 100% of the CV values are below the 15% threshold in STD mode as opposed to UHR mode.

As a point of comparison, we observed on scans of phantom 2 acquired in standard (STD) mode that 100% (93/93) of the radiomics features were stable over 10 different acquisitions for the five sets of reconstruction parameters (Figure 3). Image acquisitions in STD mode were conducted with a tube voltage of 120 kV, a collimation of 144 × 0.4 mm, a spatial resolution of 0.32 × 0.24 × 0.24 mm, and a CDTIvol of 9 mGy.

The comparison of proportions between the fractions of stable radiomics features observed in images of phantom 1 and phantom 2 demonstrated that we obtained a higher proportion of stable radiomics features when analyzing the “dry sausage” images compared to analyzing the “sponge in water” images. This difference was statistically significant for four out of five sets (sets 1, 2, 4 and 5) of reconstruction parameters (*p*-values < 0.05). While not statistically significant (*p*-value = 0.0662), we still observed a larger fraction of stable radiomics features in images of phantom 2 (vs. phantom 1) for images reconstructed with set 3 of the reconstruction parameters (Table 2).

### 3.2. Multiple Regression

For phantom 1 (sponge in water), the multiple regression led to the model described by Equation (2):

(2) $$\hat{Y}=71.275+0.550\times X_{1}-1.550\times X_{2}-3.475\times X_{3}$$

The *p*-values of the intercept, the coefficient for *X*_1_, the coefficient for *X*_2_, and the coefficient for *X*_3_ were respectively equal to 0.0107, 0.5000, 0.2171, and 0.1314. The R-squared value was high (equal to 0.9608). As only high *p*-values (>0.05) were observed, we can conclude that none of the three selected reconstruction parameters had a significant impact on the observed fraction of stable radiomics features in images of phantom 1.

For phantom 2 (dry sausage), the multiple regression led to the model described by Equation (3):

(3) $$\hat{Y}=105.400+8.600\times X_{1}-0.000\times X_{2}-17.200\times X_{3}$$

The *p*-values of the intercept, the coefficient for *X*_1_, the coefficient for *X*_2_, and the coefficient for *X*_3_ were respectively <0.0001, <0.0001, equal to 0.9046, and <0.0001. The R-squared value was high (equal to 1.0000). As small *p*-values (<0.05) were observed for both *X*_1_ and *X*_3_, we can conclude that the convolution kernel and the mono-energy value had a significant impact on the observed fraction of stable radiomics features in images of phantom 2.

For both phantoms, we observed that the highest number of stable radiomics features was achieved when images were reconstructed with the reconstruction parameters of set 1 (Br36, QIR 4, 1024 × 1024, 60 keV) (Table 2). Set 5 led to the second-highest number of stable radiomics features. For both phantom 1 (sponge in water) and phantom 2 (dry sausage), we performed a comparison of the proportions of stable radiomics features obtained from images reconstructed with set 1 and with set 5 of the reconstruction parameters (Table 5). For phantom 1, the difference in the proportion of stable radiomics features was not statistically significant between set 1 and set 5 (*p*-value = 0.8711), while the difference was statistically significant for phantom 2 (*p*-value < 0.05).

## 4. Discussion

This phantom study aimed to identify a combination of parameters offering stable reproducible radiomics signals on images generated on a photon-counting detector CT-scan. The higher proportion of stable radiomics features (80–98%) observed when analyzing the “dry sausage” (phantom 2) images compared to the “sponge in water” (phantom 1) images (67–70%) comes probably from the higher sensitivity of phantom 1 to different sources of motion during image acquisition. As we considered the same CT slice over the 10 acquisitions of each phantom, any phantom motion between acquisitions would introduce some variability of the textural information in the considered CT slice. Reviewing these 10 slices of Phantom 1 showed significant changes in image content. We identified the following factors as root causes of these changes: (1) sagging with time of the sponge in the bag filled with water, (2) deformation of the phantom in the direction of the CT bed movement, as well as perpendicular to this direction, (3) appearance or migration of air bubbles. Reviewing the 10 slices of Phantom 2 showed more image content stability due to the phantom’s relative rigidity. As those observed image variabilities mainly affected small details in the CT slices, we can infer this is the reason why most first-order radiomics features remained stable in images of both phantoms, because they dealt with large-scale image variations. These results align with other studies particularly addressing the effect of breathing motion on radiomics analysis in lung nodules, where breathing amplitude and frequency were limiting factors [25,26]. One way of surpassing the motion factor could be an exhaustive segmentation of the region of interest, ideally in three dimensions. Parmar et al. [27] did show that radiomic features stability was indeed more robust in 3D than in 2D.

This motion factor affecting the stability of radiomics features would have been stronger in standard CT scanners compared to photon-counting scanners where the motion artifact is significantly reduced due to the reduced time of acquisition [28].

The multiple regression on the fraction of stable radiomics features as a function of convolution kernel, reconstruction matrix size, and mono-energy value demonstrated that none of the three selected reconstruction parameters had a significant impact on the observed fraction of stable radiomics features in images of phantom 1 (sponge in water). This is most probably the result of phantom motions during and between the 10 scans that led to a poor fraction of stable radiomics features hiding the impact of reconstruction parameters on the informational content of the images. On the other hand, with phantom 2 (dry sausage), which was less sensitive to motions during the image acquisitions, it was possible to identify the convolution kernel and the mono-energy level as two reconstruction parameters having a significant impact on the larger fraction of stable radiomics features. We observed a statistically significant difference in the number of stable radiomics features when using set 1 of the reconstruction parameters in phantom 2 (while no statistically significant difference was observed for phantom 1). Therefore, we would recommend using Br36 as the convolution kernel and 60 keV as the mono-energy reconstruction level when performing radiomics analysis of images acquired in UHR mode. As Br36 is a smoother convolution kernel compared to Br40, we may infer that higher smoothing of images acquired in UHR mode (which involves more details and is prone to a higher level of noise) is beneficial to radiomics feature stability. Similarly, a mono-energy level of 60 keV will lead to a higher CNR (contrast-to-noise) ratio compared to 40 keV, which is also beneficial to radiomics feature stability, as shown in Euler et al. [29]. For the same phantom 2, we observed that images acquired in STD mode led to 100% stable radiomics features, confirming the negative impact of additional details and noise level on the stability of radiomics features. This aligns with a recent study done by Zhang et al. on PCD-CT where parameters that affected voxel size such as slice thickness had an influence on radiomics stability and robustness [18]. It is important to remember that this choice of parameters may impact radiomics analysis but had no impact in terms of image preference for the clinical readers.

One of the limitations of this study is that it focuses on the stability of radiomics features on a PCD-CT scan, overlooking other important aspects of radiomics, such as its discriminative power, which is also influenced by imaging parameters as described by Jimenez-del-Toro et al. [30,31]. In fact, the focus on the stability of radiomics features aims to establish a set of acquisition parameters for future clinical studies in oncology patients, emphasizing prognosis over diagnosis. Another limitation would be the selection of reconstruction parameters based on perceived image quality, which was an approach that was used to reduce the dimensionality of our image-reconstruction parameter search space. Although expert readers evaluated the image quality, some degree of subjectivity and lack of consensus are considered a limitation to this study. On the other hand, it is not straightforward that the best-perceived image quality will lead to the more appropriate image rendering for radiomics analysis. It would be interesting to extend this optimization approach to a complete parameter space independent of reader preference to make sure we finally use the best reconstruction parameters, ensuring the most reliable radiomics features.

## 5. Conclusions

Based on our experiments comparing images acquired of two different phantoms on photon-counting CT scans in high resolution, we can conclude that the higher the sensitivity of an imaged object to motion, the lower the number of stable radiomics features. Patient or organ motion will introduce a lack of reliability in a significant number of radiomics features (up to 30%). We can also conclude that thinner slices have a greater negative impact on the stability of radiomics features. Studying radiomics on photon-counting CT-scan images offers both reduced motion artifacts and high-resolution images. Using the optimal reconstruction parameters the PCD-CT could offer a stable radiomic signal, potentially serving as a biomarker.

## Figures and Tables

**Table 3:** Inputs of the multiple regression for phantom 1 (sponge in water).

Inputs	*Y* Stable Features	*X*_1_ Kernel	*X*_2_ Matrix	*X*_3_ KeV
	Num value %	1: Br36	1: 1024 × 1024	1: 60 keV
		2: Br40	2: 512 × 512	2: 40 keV
**SET 1**	69.90%	1	1	1
**SET 2**	66.70%	1	1	2
**SET 3**	67.70%	1	2	2
**SET 4**	66.70%	2	1	2
**SET 5**	68.80%	2	2	2

**Table 4:** Inputs of the multiple regression for phantom 2 (dry sausage).

Inputs	*Y* Stable Features	*X*_1_ Kernel	*X*_2_ Matrix	*X*_3_ KeV
	Num value %	1: Br36	1: 1024 × 1024	1: 60 keV
		2: Br40	2: 512 × 512	2: 40 keV
**SET 1**	96.80%	1	1	1
**SET 2**	79.60%	1	1	2
**SET 3**	79.60%	1	2	2
**SET 4**	88.20%	2	1	2
**SET 5**	88.20%	2	2	2

**Table 5:** Comparison of proportions of stable radiomics features between phantom 1 (sponge) and phantom 2 (sausage) for the 5 sets of reconstruction parameters in UHR mode.

	OBSE RVE D VALU ES		Comparison of Proportions	
**SET 1**	Sponge	Sausage	Difference	26.9%
stable	65	90	95% CI	16.6952% to 37.0802%
% stable	69.9%	96.8%	Chi—squared	24.116
total	93	93	DF	1
			Significance level	*p* < 0.0001
**SET 2**	Sponge	Sausage	Difference	12.9 %
stable	62	74	95% CI	0.1432% to 25.1300%
% stable	66.7%	79.6%	Chi—squared	3.919
Total	93	93	DF	1
			Significance level	*p* = 0.0478
**SET 3**	Sponge	Sausage	Difference	11.9 %
stable	63	74	95% CI	−0.7819% to 24.1052%
% stable	67.7%	79.6%	Chi—squared	3.375
Total	93	93	DF	1
			Significance level	*p* = 0.0662
**SET 4**	Sponge	Sausage	Difference	21.5%
stable	62	82	95% CI	9.5669% to 32.7836%
% stable	66.7%	88.2%	Chi—squared	12.241
Total	93	93	DF	1
			Significance level	*p* = 0.0005
**SET 5**	Sponge	Sausage	Difference	19.4 %
stable	64	82	95% CI	7.6384% to 30.6222%
% stable	68.8%	88.2%	Chi—squared	10.314
Total	93	93	DF	1
			Significance level	*p* = 0.0013

## Data Availability

The data presented in this study are available on request from the corresponding author. The data are not publicly available as they are intended solely for research purposes upon reasonable request.

## Abbreviations

The following abbreviations are used in this manuscript:

Photon counting detector CT-scan	PCD-CT
Ultra high resolution	UHR
Standard mode	STD
